# Molecular Cloning and Sequence Analysis of a Novel P450 Gene Encoding CYP345D3 from the Red Flour Beetle, *Tribolium castaneum*

**DOI:** 10.1673/031.008.5501

**Published:** 2008-10-06

**Authors:** Hong-Bo Jiang, Jin-Jun Wang, Guo-Ying Liu, Wei Dou

**Affiliations:** ^1^ Key Laboratory of Entomology and Pest Control Engineering, College of Plant Protection, Southwest University, Chongqing 400716, P. R. China; ^2^ Sichuan Entry-Exit Inspection and Quanrantine Bureau, Chengdu 610041, P. R. China

**Keywords:** cytochrome P450, sequence analysis

## Abstract

A novel cDNA clone encoding a cytochrome P450 gene has been isolated from the insecticide-susceptible strain of the red flour beetle, *Tribolium castaneum* (Herbst) (Coleoptera: Tenebrionidae). The nucleotide sequence of the clone, designated CYP345D3, was determined. The cDNA is 1554 bp in length and contains an open reading frame from base pairs 32 to 1513, encoding a protein of 493 amino acid residues and a predicted molecular weight of 57466 Daltons. The putative protein contains the classic heme-binding sequence motif FxxGxxxCxG (residues 430–439) conserved among all P450 enzymes as well as other characteristic motifs of the cytochrome P450s. Comparison of the deduced amino acid sequence with other CYP members shows that CYP345D3 shares 91% identity with the previously published sequence of CYP345D1 from the *T. castaneum* genome project and the nucleotide sequence identity between them is less than 80%. Phylogenetic analysis of amino acid sequences from members of various P450 families indicated close phylogenetic relationship of CYP345D3 with CYP6 of other insects than those from mammals and amore distant relationship to P450 from other families. CYP345D3 was submitted to GenBank, accession number EU008544.

## Introduction

P450 enzymes (mixed function oxidases, cytochrome P450 monooxygenases), one of the most important enzyme systems involved in insecticide detoxification or activation, are a complex family of heme containing enzymes found in most organisms. To date, various kinds of P450 enzymes have been reported in animals, microorganisms, and plants, and classified into more than 36 families (Nelson et al. 1993; Zhou et al. 2002). P450 enzymes bind molecular oxygen and receive electrons from NADPH to introduce an oxygen atom to the substrate. In insects, the diverse functions of P450 enzymes range from the synthesis and degradation of ecdysteroids and juvenile hormones to the metabolism of xenobiotics (Andersen et al. 1994; Feyereisen 1999). P450 enzymes play important roles in adaptation of insects to toxic compounds in their host plants and are involved in metabolism of all commonly used insecticides. Also, they metabolize organophosphorus insecticide compounds to more active toxicants by activation of a P=S bond to a P=O bond (Feyereisen 1999; Sams et al. 2000; Scott and Wen 2001). However, in general, P450 enzymes mediate metabolic detoxification of other insecticides.

Nomenclature of P450 genes has been established to designate all gene members of the P450 super-family with a *CYP* prefix, followed by a numeral for the family, a letter for the subfamily, and a number for the individual gene (Nelson et al. 1996; Feyereisen 1999). This system defines that members of a family share >40% identity in amino acid sequence, and members of a subfamily share >55% identity (Feyereisen 1999).

The first insect P450 gene (CYP6A1) was isolated from an insecticide-resistant strain of the housefly, *Musca domestica* (Feyereisen et al. 1989). Subsequently, many P450 genes were cloned. More than 1958 sequences of P450 genes have now been registered in the GenBank database. In China, research on the molecular cloning aspect of P450 genes started recently. In *Helicoverpa zea*, a xanthotoxin-inducible cytochrome P450 cDNA (CYP6B8) was isolated (Li et al. 2000). Three new full-length cDNAs were cloned from *Aedes albopictus* (Zhou et al. 2001) and the full length of CYP6BF1 was also obtained from *Plutella xylostella* through the SMART (Switching Mechanism At 5′ end of the RNA Transcript) technique (Li et al. 2005). Nine CYP4 fragments from *Culex pipiens* Pallens (Teng et al. 2004), two CYP6 (Ai et al. 2004a) and ten CYP4 (Ai et al. 2004b) fragments from a susceptible strain of *Helicoverpa armigera* were cloned. In addition, two new P450 cDNA fragments were gained from a deltamethrin resistant strain of *Musca domestica* using the differential display PCR technique (Ma et al. 2005).

The red flour beetle, *Tribolium castaneum* (Herbst) (Coleoptera: Tenebrionidae), is a cosmopolitan and destructive pest of raw and processed cereal grains (Sokoloff 1977; Sinha and Watters 1985; Mills and Pedersen 1990). Direct feeding on the host grain enhances mould growth and excretion of hydroxyquinone compounds contaminates and causes damage to the grain. For more than 40 years, this species has shown its ability to develop resistance to insecticides and resistant strains have spread geographically, so that it remains as one of the major pests of stored products (Assié 2007). Occurrence of resistance to insecticide such as phosphine (PH3) in many strains of *T. castaneum* was reported from many countries in the 1970s (Champ and Dyte 1976). In this study, we undertook efforts to clone the P450 genes because there is evidence that P450 enzymes might be involved in some of the resistance (Miller et al. 1999). Here, we report a novel P450 gene (CYP345D3) from *T. castaneum*.

## Materials and Methods

### Insects

The PH3 susceptible strain of *T. castaneum* was from the Entomology Laboratory of Queensland Department of Primary Industry, Australia. The insects were cultured in the Key Laboratory of Entomology and Pest Management, Southwest University, Chongqing. The beetles were reared at 30 °C in whole wheat flour fortified with 5% (v/v) Brewer's yeast under standard conditions (Beeman and Stuart 1990). The last instar larvae were collected using a sieve to separate the insects from the medium.

### Isolation of total RNA and synthesis of first strand cDNA

Total RNA for the amplification of cDNA fragments and the Rapid Amplification of cDNA Ends (RACE) were isolated from the last instar larvae of *T. castaneum* using TRNzol Reagent (Tiangen, www.tiangen.com). Twenty individuals were homogenized with at least 1 ml TRNzol Reagent in the glass homogenizer. The process of total RNA extraction and purification was carried out following the manufacturer°s instructions including a DNase (Takara, www.takara-bio.co.jp) treatment. Finally the total RNA (A260/A280= 1.8) was dissolved in 40 ***µ***l DEPC treated H_2_O and stored at -80 °C. The first strand cDNA was synthesized using 2 ***µ***g of DNase-treated total RNA by RevertAid™ First Strand cDNA Synthesis Kit (Fermentas Life Sciences, www.fermentas.com) with oligo(dT)-adaptors. The total volume of reverse transcriptional system was 25 ***µ***l. The reaction condition was performed according to the manufacturer's instructions and the reaction mixture was stored at -20 °C.

### Degenerate primers and amplification of cDNA fragment

The degenerate primers used in PCR were designed as described by Kasai et al. (1998) and the nucleotide sequences of synthetic primers were as follows: 5′-CGGARACNHYNMGNAARTAYCC-3′ for the forward primer (DP1) and 5′-CGGGNCCNKCNCCRAANGG-3′ for the reverse primer (DP2). Degenerate PCR was conducted with TGradient PCR Thermal Cycler (Biometra, www.biometra.de) using rTaqTM polymerase (Takara). There was 2 ***µ***l cDNA in the total volume of 25 ***µ***l. The PCR program included an initial denaturation step of 3 min at 94 °C and then 30 cycles were run as follows: 94 °C for 30 sec, 50 °C for 30 sec and 72 °C for 45 sec with a final extension of 10 min at 72 °C. The PCR products were separated by 1.5 % agarose gel electrophoresis and stained with ethidium bromide (EB). The band of the expected size (243 bp) was excised and the fragment was recovered with the Gel Extraction Mini Kit (Watson Biotechnologies, Inc. Shanghai).

### Cloning and sequencing of cDNA fragment

The purified 243 bp fragment was cloned into a pMD-18-T vector (Takara). After transformation into JM109 competent cells (Takara), the DNA inserts of the recombinant clones were amplified by PCR with degenerate primers used above, and sequenced in both directions (Invitrogen Life Technologies, www.invitrogen.com).

### Rapid amplification of cDNA ends (3′ RACE and 5′ RACE)

The 3′ RACE was performed using the 3′-Full RACE Core Set Ver. 2.0 (Takara). The 3′ RACE adaptor primer 3AP (anti-sense): 5′-CTGATCTAGAGGTACCGGATCC-3′ and a genespecific primer 3GSP1 (sense): 5′-TTGAACCGGAAGTCAGATGTA-3′ were used for PCR with PrimeSTAR™ HS DNA polymerase (Takara) under the following conditions: an initial denaturation at 98 °C for 5 min, followed by 30 cycles of 98 °C for 10 sec, 55 °C for 20 sec and 72 °C for 1 min and a final extension at 72 °C for 10 min. A nested PCR was conducted under similar conditions using 1 ***µ***l of the first PCR product as template, the same 3′RACE adaptor primer and another gene-specific primer, 3GSP2: 5′CCTGAACGGTTTAGTGATGAG-3′. The PCR product was excised and sub-cloned into a pMD-18-T vector. Several recombinant clones were identified by PCR amplification with 3AP and 3GSP2 and then sequenced as described above.

The 5′ RACE was conducted with BD SMART™ cDNA Amplification Kit (Clontech, www.clontech.com). The 5′ region was amplified using a gene-specific antisense primer 5GSP1: 5′-CCTTCCCCAAATGGCAAATACG-3′ and a 5′ RACE adaptor primer, UPM: 5′-CTAATACGACTCACTATAGGGCAAGCAGTGGTATCAACGCAGAGT-3′. The PCR amplification was performed with Advantage™ 2 PCR Kit (Clontech) under the following conditions: an initial denaturation at 94 °C for 3 min, Mowed by 30 cycles of 94 °C for 30 sec, 65.5 °C for 30 sec and 72 °C for 2 min and a final extension at 72 °C for 10 min. Again, a nested PCR was conducted using 1 ***µ***l of the first PCR product as template, the 5′ adaptor primer NUP: 5′-AAGCAGTGGTATCAACGCAGAGT-3′ and the gene-specific primer 5GSP1 used above. The PCR condition was identical with that described above. The PCR product was excised and sub-cloned as described above.

Amplification of full-length cDNA was confirmed with NUP and FGSP1: 5′GAAAGTTTCGAAAATTGTGTG-3′. The cDNA in 5′ RACE was used as template. The PCR amplification was performed with Advantage™ 2 PCR Kit (Clontech) under the following conditions: an initial denaturation at 94 °C for 3 min, followed by 30 cycles of 94 °C for 30 sec, 55 °C for 30 sec and 72 °C for 2 min and a final extension at 72 °C for 10 min. All the gene-specific primers used in 3′ RACE and 5′ RACE was designed utilizing Primer Premier 5.0 (http://www.PremierBiosoft.com).

### Sequence analysis

DNA sequence was determined using the ABI-PRISM 3730 sequencer (Invitrogen). Searching of similar sequences was performed using BlastP in the non-redundant protein sequences (nr) database of the NCBI website (http://www.ncbi.nlm.nih.gov). A phylogenetic tree was constructed by MEGA version 3.1 (Kumar et, al. 2004) using the method of Neighbor-Joining.

## Results

### cDNA and deduced amino acid sequence of the CYP345D3

**Figure 1.**
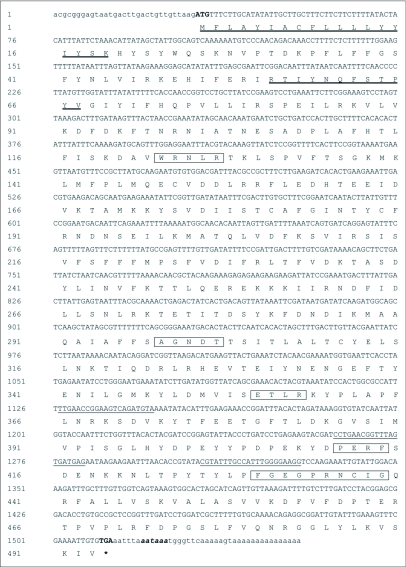
Nucleotide and deduced amino acid sequence of CYP345D3. The nucleotides underlined show the positions of gene specific primers used in the experiment. The start codon ATG is indicated with bold and the stop codon TGA is indicated with bold and by an asterisk. Polydenylation signal AATAAA is shown in bold italic lowercase letters. The heme-binding sequence motif FxxGxxxCxG and other sequence motif are indicated by the boxed amino acids. The transmembrane domains are shown in deeply underlined amino acid residues.

**Figure 2.**
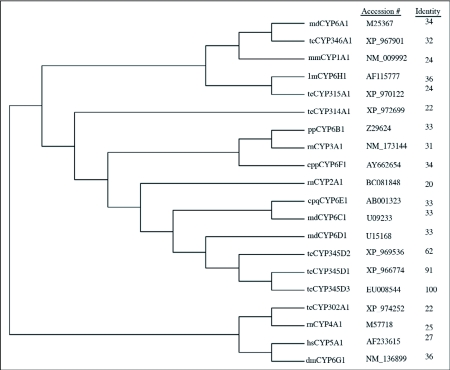
Phylogenetic relationship based on the amino acid sequence comparisons of Cytochrome P450s from various CYP families. GenBank accession numbers are shown followed the P450s' name. Identity is obtained by pairwise alignment of amino acid sequence of CYP345D3 with indicated P450s. Letter designation: cpp, *Culex pipiens pollens*; md, MUSCCJ *domestica*; cpq, *Culex* *pipiens quinquefasciatus*; lm, Locusta *migratoria*; tc, *T. castaneum*; dm; *Drosophila melanogaster*; pp, *Papilio polyxenes*; rn, *Rattus norvegicus*; hs, *Homo sapiens*; mm, *Mus musculus.*

The full-length cDNA sequence with the deduced amino acid sequence below the nucleotide sequence (GenBank accession number, EU008544) is shown in Figure 1. The cDNA sequence was 1554 bp in length with an open reading frame of 1479 bp encoding 493 amino acid residues and had a predicted molecular weight of 57466 Daltons. The putative polydenylation signal AATAAA is shown in bold italic lowercase letters. The deduced protein sequence shares high identity (91% and 62%, respectively) to probable CYP345D1 and CYP345D2 (GenBank accession number, XP_966774 and XP_969536) from *T. castaneum*. Identity to other members of P450 families is shown in Figure 2. According to the alignment, the heme-binding sequence motif FxxGxxxCxG (residues 430–439) that is universal among P450 enzymes is found in the deduced amino acid sequence. After submission to the P450 nomenclature committee, the sequence was classified into a new family from *Tribolium* called CYP345, and it was named CYP345D3 using the nomenclature of Nelson et al. (1996) and Nelson (2006).

### Phylogenetic relationship with other P450 families

The deduced amino acid sequence of CYP345D3 contains all important motifs characteristic of the P450 enzymes, particularly the CYP6 family. Using MEGA version 3.1 software (Kumar et, al. 2004), a phylogenetic tree was constructed using the Neighbor-Joining method (Figure 2). The CYP345D3 sequence was found to be more closely related to CYP345D1 and CYP345D2 from *T. castaneum* and other insect CYP6 members than families of CYP1A1, CYP2A1, CYP3A1, CYP4A1, CYP5A1 from mammals and CYP302A1, CYP314A1, CYP315A1, CYP346A1 from *T. castaneum.* Not surprisingly, phylogenetic analysis of amino acid sequences from members of various P450 families indicated closer phylogenetic relationship of CYP345D3 with CYP6 members from *T. castaneum* and other insects than from mammals and a more distant relationship to P450s from other families.

## Discussion

Gene cloning by PCR using degenerate primers derived from conserved amino acid sequences from other species has proven to be a powerful method to obtain related DNA sequences from the target species. Although the P450 super-family has a very divergent sequence and the overall homology maybe less than 40% even within the same family, particular in insects (Wang and Hobbs 1995), there are some function-critical sequence motifs preserved during evolution. Typically, the sequence features of P450 genes can be listed as follows. First, the classic heme-binding sequence motif FxxGxxxCxG (residues 430–439) that is conserved among all P450 enzymes (Ranasinghe and Hobbs 1998; He et al. 2002; Nelson 2006), is found in the deduced amino acid sequence, the cysteine of which is present in all P450 sequences. Secondly, the sequence motif (A/G) GxxT (residues 298–302) corresponds to an I-helix which is a conserved alpha helical region in proposed three dimensional models for a member of P450 protein based on the known structure of several bacterial enzymes (Von Wachennfeldt and Johnson 1995). The “T” (Threonine, Thr) is part of the molecular oxygen-binding site (Nelson 2006). Site directed mutagenesis studies have suggested that this highly conserved region around Thr302 could be involved in oxygen binding, while some of the hydrophobic residues preceding the Thr could be involved in substrate binding (Shimizu et al. 1989). Thirdly, the motif WxxxR (residues 123–127) corresponding to a C-helix sequence and conserved in most eukaryotic P450s has been found. Furthermore, the K-helix ExxR sequence motif (residues 355–358), the best conserved feature in P450s, and salt bridge formation in the mature protein has also been reported (Graham-Lorence and Peterson 1996; Nelson 2006). Additionally, the PERF (residues 411–414) sequence motif which is part of the meander before the heme thiolate ligand (Peterson and Graham-Lorence 1995) is present. Analysis with the SOSUI program (http://www.hgsc.bcm.tmc.edu, SearchLauncher, Human Genome Center, Baylor College of Medicine, Houston, TX) for protein secondary structure prediction (Hirokawa et al. 1998) indicates that the CYP345D3 is a membrane protein with two transmembrane domains located near the N-terminus. The primary transmembrane domain contains amino acid residues 1–19 and the secondary one contains residues 56–78. These highly hydrophobic regions serve as the signal peptide for cotranslational insertion of the protein into the target membrane appear in most P450 enzymes (Pernecky and Coon 1996). These results reveal that CYP345D3 contains characteristic functional domains for P450 enzymes.

The deduced amino acid sequence of CYP345D3 shares 91% identity with probable CYP345D1 as computed from the genome of *T. castaneum*, while the nucleotide sequences share less than 80% identity with each other. However, the CYP345D3 sequence is not present in the *Tribolium* assembly from the genome project. This suggsts that the *Tribolium* species are highly polymorphic, and the strain used here is different than that used in the genome project. Analysis of the CYP345D3 sequence, revealed the conserved functional sites. However, the real function of the sequence is still unknown.

Molecular cloning of CYP345D3 is just the first step to study the cytochrome P450 system in the red flour beetle, which has not been very well studied relative to their detoxification of insecticides. In future research, heterogenous expression of CYP345D3 such as in *Escherichia coli* and *Pichia postoris* can be used to identify the biochemical characteristics of this enzyme and validate the function of it. In conclusion, the isolation of the CYP345D3 sequence provides some basic knowledge to understand the P450 enzyme system in *T. castaneum*. However, better understanding of the system needs much further study.
